# Supplementary material for: Characterization of biliary microbiota dysbiosis in extrahepatic cholangiocarcinoma
Source: PLoS One. 2021 Mar 9;16(3):e0247798. doi: 10.1371/journal.pone.0247798 (PMC7943025; doi:10.1371/journal.pone.0247798)
Supplement: Attachment — Submitted filename: Response to Reviewers.pdf [file pone.0247798.s005.pdf]

Pradeep Dudeja
Academic Editor
PLOS ONE

Dear Editor Pradeep Dudeja,

Subject: Submission of revised paper CHARACTERIZATION of biliary microbiota dysbiosis in extrahepatic cholangiocarcinoma (PONE-D-20-20572).

We would like to thank you for the consideration brought our paper, and for allowing us to resubmit a modified version of our manuscript to PLOS One. After careful revision, we responded in a "point-by-point manner" to your own comments as well as to the reviewers' remarks. The new version has been revised by an English native lecture. We thank reviewers for their fruitful comments. One of reviewers recommended adding a mechanistic approach in the present study. We wish to stress out that several studies around main hypotheses are already in progress. Up to know there is no evidence which out of pathways we investigate is mainly involved in the carcinogenesis. This the reason why we decided including new data regarding tumor staging and long term follow up of patients. Unfortunately, we didn't find any predictive value in bacterial changes for estimating prognosis likely due to the small size of our cohort cases. We are confident that our study needs to be reproduced through various ethnic populations before going more in-depth through one of these pathways.

We also would thank you for the additional time granted to prepare this present modified version. Consequently, the following items are considered in the revised manuscript:

- Responses to each point raised by the academic editor and reviewer(s).
- A marked-up copy of our manuscript that highlights changes made to the original version and identified as 'Revised Manuscript with Track Changes'.
- An unmarked version of revised paper without tracked changes identified as 'Manuscript'.

Sincerely

M. Saab, I. Sobhani

Journal Requirements: responses are highlighted using color

When submitting your revision, we need you to address these additional requirements.
1. Please ensure that your manuscript meets PLOS ONE's style requirements, including those for file naming. The PLOS ONE style templates can be found at
We have carefully reexamined the manuscript to meet the journal's style requirements.

2. Thank you for your ethics statement : 'All patients were enrolled at Firoozgar Hospital (Teheran) for thepresent translational study and provided their informed consent to conduct microbial analyses on the biliary juice recovered during the ERCP (Ethics Committee approval under ID: IR.IUMS.REC.1397.115 of Iran university of medical sciences).'

(a) Please amend your current ethics statement to confirm that your named institutional review board or ethics committee specifically approved this study.

As stated in the first version, the name of the institution (Firoozgar Hospital) and the related ethical committee has granted approval registered under n° ID: IR.IUMS.REC.1397.115 of Iran University of medical sciences. These details are now included in page 6 of the revised manuscript, line 127 to 130.

(b) Once you have amended this/these statement(s) in the Methods section of the manuscript, please add the same text to the "Ethics Statement" field of the submission form (via "Edit Submission").
For additional information about PLOS ONE ethical requirements for human subjects research, please refer to http://journals.plos.org/plosone/s/submission-guidelines#loc-human-subjects-research."

Details related to the ethics statement is also included in the submitted revised manuscript page 6, L131-139

3. Please provide additional details regarding participant consent. In the ethics statement in the Methods and online submission information, please ensure that you have specified what type of consent you obtained (for instance, written or verbal, and if verbal, how it was documented and witnessed). If your study included minors, state whether you obtained consent from parents or guardians.

After full explanation of the aim of the study and the process by a physician, written consent was signed by each enrolled patient and documents are held by the Iranian partner for 15 years. All participants were adult with very well state of intelligence.

It was a prospective study and patients were consecutively enrolled. This is now indicated in page 6 of the revised manuscript, line 131 to 133.

4. In your Methods section, please provide additional information about the participant recruitment method and the demographic details of your participants. Please ensure you have provided sufficient details to replicate the analyses such as: a) the recruitment date range (month and year), b) a description of any inclusion criteria that were applied to participant recruitment and c) a description of how participants were recruited.

The inclusion criteria were: Adult (age more than 21), suspected of biliary pathologies such as elevated liver enzymes and Total bilirubin, presence of stricture in Endosonography or MRCP.

Exclusion Criteria were: history of viral hepatitis, metabolic hepatitis, Autoimmune hepatitis, Alcoholic hepatitis, NASH, medication induced hepatitis, chemotherapy.

Because of the rarity of CCA and the difficult laboratory confirmation we have enrolled all suspected patients during 15 months for case selection. One hundred out of them had confirmed CCA and were followed up. Biliary juice has been obtained and has been collected in GILDRC (center for biobank intestinal liver diseases collection) and clinical data (including demographic information) and date of sampling have been registered .We added all these details to the Methods section of the revised manuscript.

5. Please provide a sample size and power calculation in the Methods, or discuss the reasons for not performing one before study initiation.

At the beginning of the study there was no published data allowing us to formulate a hypothesis based on the expected difference between cases and controls. Thus, we planned to enroll at least n=30 individuals in each (cases, controls) subgroup. Once 30 CCA cases have been included the study was ended. As expected during the study period more controls than cases have been included. We should emphasize that two out of 30 CCA cases and 3 out of 50 controls have been excluded due to lack of materials. Finally, 28 CCA cases could be compared to 47 controls in an intention to analyze strategy.

6. To comply with PLOS ONE submission guidelines, in your Methods section, please provide additional information regarding your statistical analyses. For more information on PLOS ONE's expectations for statistical reporting, please see https://journals.plos.org/plosone/s/submission-guidelines.#loc-statistical-reporting.

We analyzed abundances using a user-friendly website for metataxonomic analysis (SHAMAN, Pasteur Institute, Volant 2020) and statistical analyses are performed with tests implemented in this website. Now we indicate in the manuscript and state clearly that our analysis can be performed by anyone using the two files provided in the supplementary information (S1 File and Table S3).

Point per point responses to Reviewers' comments:

Reviewer #1: • Major comments:
i. Data is held at institute and "may" be accessible under specific request is not adhering to the journals requirements to make all data underlying findings available online, especially since this is large data that could potentially lead to incredible findings in the fields of microbiome and CCA research.

We agree that all data including row and FastQ data should be shared. However, we gave all outputs under supplementary data all outputs. We accept sharing FastQ data in order to render results accessible to the readers of your journal, once the manuscript is accepted for publication.

ii. The authors claim no financial support for this work, however, this manuscript demonstrates intensive work.

We point out that we have not obtained any specific financial support from Iranian university or authorities for the clinical part. All charges for performing DNA extraction and sequencing were supported by UPEC Université Paris Est Creteil, EC2M3 Lab's budget according to the main program of our group which is focused on the markers related to digestive tumors. This program and the related budgets are revised and evaluated every 5 years.

iii. Wald test is performed for the differential abundance analysis, but due to small sample size <30 of CCA, it is recommended that likelihood ratio test be performed.

We did not perform a Wald test per se. We used the website SHAMAN (Vollant 2020) that implement statistical analysis based on the DESeq2 R package which robustly identifies the differential abundant genera using the Generalized Linear Model approach. The statistical models implemented in this program takes in account small size samples.

iv. There is no information on the severity of CCA in patients (or early/late stage PSC/IBD/etc in cases of co-morbidities) and there is no information on ethnicity or race on patients studied. The authors should attempt to present this information for transparency.

We should mention French law do not allow screening racial characteristics; this data will not be available except the information that all patients have been enrolled in Iran. Thus, we can speculate that patients' ethnicity and race were homogeneous since all have been enrolled from the same Iranian hospital. We also included now follow-up of patients. During 15 months

follow-up, 16 patients died due to cancer complications. Ten out of them were males. The severity of CCA such as Tumour staging according to the international clinic are now included in Table 1. We should indicate that all co morbidities have been included in Table 1 (first version) and Table 1 and Table S3 (present revised version)

v. Methods in this manuscript should be applied to established data sets, especially those cited in the discussion, to verify that similar results are found. This manuscript claims to characterize CCA bile microbiome but does not verify its findings on publicly available data or own previously published data sets. Also, there is no comparison of microbiome from CCA-No to those of other biliary diseases or duodenal samples which may demonstrate the true novel findings of this manuscript and reduce suspicion of collection method contamination.

We should emphasize that we characterize in this study the biliary microbiota and not the microbiome. The biliary microbiota characterization was performed with routine precaution applied to pathobionts and/or nosocomial multi-resistant strains isolated from the biliary duct in an academic hospital. Although we cannot rule out contamination with duodenal milieu, this possibility appears unlikely because recovery of biliary effluent is routinely performed after bile duct cannulation under endoscopy. However, to satisfy the reviewer, we now chose the expression “effluent through ERCP exam” instead of “biliary milieu” as well as in Results and Discussion sections.

In the revised manuscript, we have compared our results with other publications. Particularly regarding the ratio of dominant bacteria in our series and in others: see pages 15 up to 18 lines 313-323; 333—342, 360-373 and 381-384 in the new version. In addition, we now included page 16 in the discussion comparison with more recent papers that overview gut microbiota and not biliary microbiota analyses: lines 336-344.

- **Minor comments:**

i. All figures say “Non” instead of “No” when indicating comorbidity existence in CCA or control patients.

We would like to thank you for your comment and reassure that the correction has been done in tables and figures: 1B, 2B and 3B “Non” has been replaced by NO.

ii. Differential abundance should be presented in linear modeling graph for data presentation to reduce confusion of the base mean and log fold change.

In order to reduce confusion, we give now both “Base mean” and “Fold Change” with related adjusted p value. Please see tables 2 and 3, in the revised manuscript.

iii. The discussion does not speculate the on the role Anoxybacillus, Geobacillus, and Meiothermus play in the biliary milieu. Additionally. There is no mention of bile altering

bacteria levels which would be a great addition to this manuscript since bile flow, bile composition and cholestasis are interrelated in CCA and other biliary diseases.

We could not speculate on these bacteria since no basic microbiological data are available. In the discussion we have now highlighted CCA-related dysbiosis in pages 15-16 lines 333-336 and speculated only on those bacteria for which basic data are available in the international literature.

iv. Introduction and discussion should be edited. There is limited information on the impact of biliary microbiome dysbiosis in CCA development or how important dysbiosis of microbiome composition is in other diseases. The detection method of CCA is not of high importance for the findings in this manuscript unless it is to address any potential contamination from method of collection (which should be reserved for discussion and not introduction).

We agree. We have deleted some details (referring to spyglass procedures sometimes necessary to verify absence of tumors through the biliary tract). However, we should mention that details are given for the detection of CCA focused on methods of biliary effluent collection according to the reviewer asking to be ensured about duodenal contamination during the collection process of biliary juice (Lines 139-144). In the introduction, we now mention, "how important dysbiosis of microbiote composition is in other diseases" (page 4, lines 58-62 first version and pages 4-5, lines 84-88 and 93-98 new version). In the discussion and conclusion in the revised version we take the attention into bias regarding the collection of the effluents: see limitation page 18, lines 387-393.

v. Figures should be combined (all figure 1 together) rather than spread out. The figure legends should be placed outside of manuscript flow since that interrupts the readers comprehension.

Figures are now combined, and captions are placed outside of the manuscript flow according to the reviewer's Request.

Reviewer #2: The current manuscript submitted by Massa Saab et al was designed to characterize biliary microbiota in consecutive patients with a histologically proven CCAe, and their results were compared to a series of patients with benign biliary affected diseases (PBBs), which were considered the control. Using 16S RNA sequencing has demonstrated significant differences in the composition of the biliary microbiota between the two populations, which could implicate CCA-associated dysbiosis in the biliary carcinogenesis.

Overall, 32% of CCA and 22% of control patients displayed another associated disease, such as diabetes, pancreatitis, inflammatory bowel disease, or primary sclerosing cholangitis. Comparisons considered associated diseases. Principal coordinate analysis (PCoA) detected a significant disparity of the biliary microbiota composition between the CCAs and controls without an associated disease. Levels of Bacteroides, Geobacillus, Meiothermus, and Anoxybacillus genera were significantly higher in CCA patients' biliary microbiota, without an associated disease, than in the controls. A specific CCA-related dysbiosis is identified as compared to controls independently from associated diseases, therefore a microorganism community might be involved in the CCA pathogenesis, as suggested by authors. It is an interesting paper and the experiments were properly performed. However, the mechanisms between biliary microbiota and CCA development/progression are undefined, and the data on biliary carcinogenesis are descriptive. Additionally isolated CCA cells need to be better characterized, to make sure that they display the CCA phenotypes associated with biliary microbiota as expected. Moreover, the data is too preliminary and lack of the significance and mechanistic studies to define the specific biliary microbiota associated cancer signaling pathways.

Comments:

1. The major issue of the current manuscript is the mechanisms between biliary microbiota and CCA development/progression are undefined, and the data on biliary carcinogenesis are descriptive. Actually several manuscripts have proved the associations among gut microbiota, bile acid metabolism and cytokines in cholangiocarcinoma development and progression. Therefore further functional characterizations for CCA development and progression are need for the current manuscript.

As far as we know up to now only two studies (*Avilés-Jiménez et al. 2016* and *Chen et al. 2019*) aimed to characterize biliary microbiota in small series, n=10 CCA in Clinical Microbiol Infection and n=8 in BioMed Research International, respectively. In addition, *Pedro Peirera et al 2017* in PlosOne studied the biliary microbiota in primary sclerosing cholangitis aimed the: Impact on disease progression and development of biliary dysplasia/Carcinoma with n=11. We consider that the total number of cases, in the literature, is not so far enough to conceive a mechanistic pattern. Since we should distinguish between intra hepatic and extra hepatic CCAs, we decided to focus on extra hepatic cases in order to identify one or several bacteria function, which might be involved in the biliary carcinogenesis. We are now undertaking additional approaches to consider mechanistic pattern in both intra as well as extra-liver CCAs. We estimate that our series is growing the cohort of patients with CCA in whom biliary microbiota is characterized.

2. The isolated/primary CCA cells from human CCA tissues were not well characterized and the authors failed to show the CCA phenotypes associated with biliary microbiota as expected. Therefore their cancer associated biliary microbiota phenotypes are questionable.

We did not report primary CCA cells in our study. We clearly showed a significant association between biliary microbiota and the diagnosis of CCA as assessed by cytology and/or histology features as routinely used in clinics. We should remind that such association constitutes the first step of cancer-related dysbiosis.

3. There is some confusion in the manuscript to prove that the levels of Bacteroides, Geobacillus, Meiothermus, and Anoxybacillus genera were significantly higher in CCA patients' biliary microbiota, without an associated disease, than in the controls. More detailed mechanistic studies should be carried out to define the specific mechanisms of malignant transformation and their down stream signaling mechanisms.

We should remind that as mentioned in the first version (page 8 and 12, lines 149-152 and 220-226) of our manuscript, all differences between CCA and Control groups have been stratified on comorbidities (for details see Table 1). We think that the reviewer's remark is out of the statistical scope. Moreover, the mechanistic pathway we might suggest would be only a speculation on how bacteria may affect biliary carcinogenesis.

4. The rationale should be provided in introduction. Summary is missing in the discussion section and the conclusion needs to be detailed.

In the revised manuscript we now indicate that "No significant data available in the literature conducted to characterize in a series of 30 extra hepatic CCA cases the biliary signature as compared to a group of patients undergoing the same procedure in whom no tumor could be detected" (page 5 lines 108-114 Introduction section). A summary is now included page 18, lines 387-393 before the conclusion section and we have detailed the conclusion page 18, lines 397-404.

5. Statistical analysis should be included in Figure 1 & 2 with some detailed CCA cancer stage and survival information.

During the 15 months follow-up period, 16 patients died due to cancer complications. Ten out of them were males with the median survival of 11 months.
Data on the cancer staging and survival information severity are now included in Table 1 and in Supplementary data, table S3 (new version). Since no significant difference has been observed regarding these new parameters, we did not modify the presentation of results and discussed briefly the interest of microbiota regarding the prognosis page 17, lines 370-374.

6. There are not enough data in the main manuscript. Therefore, the supplementary information should be moved to the main text.

We tried fitting all date within an optimal size of manuscript according the journal's requirement. We are willing to include all our figures in the main corpus of manuscript if requested by the Editor.

7. English writing skills need to be improved. The format of the fonts and some technical terms should be consistent.

We enclose here the contact details with the contribution office for native English (CREMER CONSULTING by Dr. Gabrielle Cremer, address 14 RUE SLEIDAN ,67000 STRASBOURG) and are somewhat embarrassed by this remark.

8. The discussion needs to be modified and focused. Some of the descriptions are disconnected from the central focus in the discussion section.

We revised the main corpus of the discussion and hope that it is has become more accurate and focused.
